# Multiple independent *MGR5* alleles contribute to a clinal pattern in leaf magnesium across the distribution of *Arabidopsis thaliana*

**DOI:** 10.1111/nph.70069

**Published:** 2025-03-24

**Authors:** Emmanuel Tergemina, Shifa Ansari, David E. Salt, Angela M. Hancock

**Affiliations:** ^1^ Department of Plant Developmental Biology Max Planck Institute for Plant Breeding Research Cologne 50829 Germany; ^2^ School of Biosciences University of Nottingham Sutton Bonington LE12 5RD UK; ^3^ Department of Botany and Plant Pathology Purdue University West Lafayette 47907 IN USA

**Keywords:** allelic heterogeneity, *Arabidopsis thaliana*, CNNM, evolution, genome‐wide association mapping, MGR5, natural variation, plant

## Abstract

- Magnesium (Mg) is a crucial element in plants, particularly for photosynthesis. Mg homeostasis is influenced by environmental and genetic factors, and our understanding of its variation in natural populations remains incomplete.
- We examine the variation in leaf Mg accumulation across the distribution of *Arabidopsis thaliana*, and we investigate the environmental and genetic factors associated with Mg levels. Using genome‐wide association studies in both the widespread Eurasian population and a local‐scale population in Cape Verde, we identify genetic factors associated with variation in leaf Mg. We validate our main results, including effect size estimates, using Clustered Regularly Interspaced Short Palindromic Repeats (CRISPR) mutagenesis.
- Our findings reveal a significant association between leaf Mg and latitude of origin. In Eurasia, we find a signal at the nutrient‐response regulator, *RAPTOR1A*, and across the species range, we find that multiple alleles of the Mg transporter, MAGNESIUM RELEASE 5 (*MGR5*), underlie variation in leaf Mg and contribute to the observed latitudinal cline.
- Overall, our results indicate that the spatial distribution of leaf Mg in *A. thaliana* is affected by climatic and genetic factors, resulting in a latitudinal cline. Further, they show an example of allelic heterogeneity, in which multiple alleles at a single locus contribute to a trait and the formation of a phenotypic cline.

## Introduction

Magnesium (Mg) is an essential nutrient in living organisms. In humans, Mg serves as a cofactor in over 300 enzymatic reactions and as a counter‐ion for ATP (Jahnen‐Dechent & Ketteler, 2012). Mg influences muscle, nerve, cardiac, and endocrine functions (Jahnen‐Dechent & Ketteler, 2012; Fiorentini *et al*., 2021). A substantial proportion of people in developed countries are at risk of Mg deficiency, partly due to the unintended consequences of the green revolution, which promoted fertilization with a combination of nitrogen (N), phosphorus (P), and potassium (K). Consequently, farmers have tended to prioritize these three‐component fertilizers to increase crop yield and growth, resulting in soils depleted in secondary elements such as Mg (Guo *et al*., 2016). However, since potassium inhibits the absorption of Mg, the standardization of NPK supplementation resulted in a widespread reduction in Mg in crops. Although plants represent the principal source of Mg for humans, Mg has received comparatively little attention in agriculture and has thus been referred to as a ‘forgotten element’ (Cakmak & Yazİcİ, 2010).

Mg deficiency in plants restricts their growth and productivity (Aitken *et al*., 1999; Guo *et al*., 2016), primarily due to its essential role in photosynthesis. This is because Mg is the central element of Chl and acts as an activator of enzymes involved in photosynthetic CO_2_ fixation (Shaul, 2002; Hawkesford *et al*., 2012). Typical plant responses to Mg deficiency include sugar accumulation in leaf tissues and leaf interveinal chlorosis on older leaves, leading to impairments in plant growth and yield (Hermans & Verbruggen, 2005; Römheld, 2012). Therefore, understanding the factors that impact Mg accumulation in plants has important implications for agriculture and human nutrition.

Mg acquisition in plants depends on its abundance and availability in soils. The abundance of Mg in soils is highly dependent on the parental material from which the soil develops and anthropogenic factors such as agricultural intensity. Mg availability depends on various edaphic factors, including soil pH, cation competition, cation exchange capacity, and environmental factors, including precipitation, temperature, and atmospheric carbon dioxide (CO_2_) levels (Mesić *et al*., 2007; Sun *et al*., 2013; Loladze, 2014). Notably, Mg binds weakly to soil colloids due to its large hydrated radius, leading to its susceptibility to leaching (Maguire & Cowan, 2002; Gransee & Führs, 2013). This susceptibility is particularly pronounced in acidic soils with reduced cation exchange capacity (Aitken *et al*., 1999; Grzebisz, 2011). In addition, high precipitation exacerbates Mg leaching, leading to low Mg availability in geographic regions with high rainfall (Mesić *et al*., 2007). Considering that climate change is inducing shifts in precipitation, temperature, and atmospheric CO_2_ levels, it is important to improve our understanding of the mechanistic relationships between these environmental factors and Mg availability, which will be critical for predicting crop responses due to global change.

Some aspects of the molecular pathways underlying Mg homeostasis are known. Mg transporters play a fundamental role in maintaining Mg homeostasis and are highly conserved across eukaryotes and prokaryotes. In prokaryotes, Mg homeostasis is maintained by CorA, CorB/C, MgtA/B, and MgtE Mg transporters (Franken *et al*., 2022). While MgtA/B and MgtE do not appear to have any close homologs in plants, the CorA and CorB/C homologs have been identified. CorA homologs in plants were discovered over 20 years ago and are known as MAGNESIUM TRANSPORTER or MITOCHONDRIAL RNA SPLICING 2 transporters (Schock *et al*., 2000; Li *et al*., 2001). By contrast, CorB/C homologs were recently revealed in plants and named MAGNESIUM RELEASE (MGR) (Meng *et al*., 2022; Tang *et al*., 2022; Zhang *et al*., 2022). The MGR family of proteins shares a transmembrane domain of unknown function 21 and Cystathionine‐β‐Synthase (CBS) domains, characteristic in humans of the CBS domain divalent cation transport mediators (CNNMs), also previously called ancient conserved domain proteins (De Baaij *et al*., 2012). Additionally, *MAGNESIUM/PROTON EXCHANGER 1* (*MHX1*), a gene encoding a proton antiporter that transports Mg, Zn, and Fe into the vacuoles in exchange for protons, may contribute to Mg homeostasis specifically in plants (Shaul *et al*., 1999). Overall, the abundance of research on *A. thaliana* provided information about several loci governing Mg homeostasis in plants.

Despite previous success in understanding nutrient homeostasis in natural populations of plants (Baxter *et al*., 2008, 2010; Arnold *et al*., 2016; Busoms *et al*., 2018; Yang *et al*., 2018; Tergemina *et al*., 2022), our knowledge of the genetic determinants behind natural variation in Mg accumulation has remained limited. In this study, we examine the environmental factors influencing leaf Mg content in the widespread Eurasian *A. thaliana* population. Then, using a genome‐wide association mapping approach in both the Eurasian population and a local population from the Cape Verde Islands (CVI), we characterize the trait's genetic architecture. We identify multiple MAGNESIUM RELEASE 5 (*MGR5*) alleles associated with Mg accumulation across the global distribution, demonstrating a case of allelic heterogeneity at *MGR5* linked to leaf Mg accumulation.

## Materials and Methods

### Plant material used for leaf Mg analysis

To analyze leaf Mg in natural populations of *Arabidopsis thaliana* (L.) Heynh., we used previously published Mg accumulation data (Campos *et al*., 2021). After retaining accessions present in both the 1001 Genomes panel (The 1001 Genomes Consortium, 2016) and the panel used by Campos *et al*. (2021), we excluded accessions with low sequence coverage (Voichek & Weigel, 2020) and potential contaminant accessions (Pisupati *et al*., 2017), resulting in a global panel of 810 accessions. Additionally, we grew 166 natural accessions from Santo Antão, CVI (Fulgione *et al*., 2022), together with Col‐0 (6909) as a control, following the experimental protocol of Campos *et al*. (2021). Seeds were stratified for 7 d in the dark at 4°C on Petri dishes with 800 μl GA4+7 (100 μM). We then sowed the seeds on jiffy‐7® pots presoaked in a solution containing Na_2_HAsO_4_·7H_2_O (0.142 μM), Cd(NO_3_)_2_·4H_2_O (5.935 μM), Co(NO_3_)_2_·6H_2_O (0.534 μM), LiNO_3_ (0.053 μM), Ni(NO_3_)_2_·6H_2_O (0.534 μM), K_2_SeO_4_ (0.427 μM), Sr(NO_3_)_2_ (98%) (0.106 μM), and RbNO_3_ (0.0774 μM). We propagated three replicates for each Santo Antão accession in a randomized block design under controlled glasshouse conditions (8 h of light, 21°C during the day, 14°C at night). We watered the plants once per week with a 0.25× Hoagland solution, which is designed to provide a balanced mix of nutrients that are essential for plant growth. The solution contained 0.25 mM NH_4_H_2_PO_4_, 1 mM Ca(NO_3_)_2_·4H_2_O, 0.5 mM MgSO_4_·7H_2_O, 1.5 mM KNO_3_, 0.075 μM CuSO_4_·5H_2_O, 0.2 μM ZnSO_4_·7H_2_O, 11.5 μM H_2_BO_3_,1.3 μM MnCl_2_·4H_2_O, 0.028 μM MoO_3_, and was supplied with 10 μM Ferric iron (FeIII) chelated with N,N‐di(2‐hydroxybenzyl)ethylenediamine‐N,N‐diacetic acid monohydrochloride hydrate (Fe(III)‐HBED). Four weeks after germination, we harvested two to three young leaves per plant for ionomic analyses. The leaves were washed three times with 18.2 MΩ.cm milli‐Q water (Merck Millipore) before being transferred to 1.5 ml Eppendorf tubes. The samples were then dried overnight at 80°C. Leaf elemental content analysis normalized by weight was performed at the Ionomics facility at the University of Nottingham using an inductively coupled plasma mass spectrometry PerkinElmer NexION 2000, as described in Campos *et al*. (2021). To account for block effects in both the 1001 Genomes and the Santo Antão panels, we estimated leaf Mg values using the best linear unbiased estimates (BLUEs) with the lme4 package (v.1.1‐35.5) (Bates *et al*., 2015) in R (v.4.4.2) (R Development Core Team, 2017).

### Environmental association analysis

To examine the association between latitude and leaf Mg content variation, we conducted a Spearman rank correlation test (spearmanr function in SciPy, v.1.6.2) (Virtanen *et al*., 2020). To identify potential abiotic drivers of leaf Mg variation, we collected environmental data for geo‐referenced *A. thaliana* accessions from the 1001 Genomes panel collected within Eurasia. We defined the Eurasian population as accessions collected between 36.52°S, 68.80°N, −8.54°W, and 38.28°E, resulting in 690 accessions. In total, we collected information for 35 environmental variables from five publicly available sources (Supporting Information Table S1). From WorldClim v.2.1 geodatabase, we collected data for 19 bioclimatic variables (derived from temperature and rainfall) and monthly averages of solar radiation (Fick & Hijmans, 2017), with a spatial resolution of 30 arc seconds (*c*. 1 km). We downloaded the aridity index, defined as the ratio of precipitation and evapotranspiration of a reference crop (ET0), at a resolution of 30 arc seconds (*c*. 1 km) from the Consortium of International Agricultural Research Centers (CGIAR) Consortium for Spatial Information (Trabucco & Zomer, 2019). We accessed data for 13 soil variables with a spatial resolution of 500 m from the European Soil Database Centre (Ballabio *et al*., 2016, 2019). Since this dataset did not contain information about Mg content, we also downloaded estimated Mg content based on mobile metal ion extractions, which is expected to reflect bioavailable Mg, with a spatial resolution of 50 km from a separate data source (Négrel *et al*., 2021). All spatial data analysis was done in Qgis (v.3.22). We extracted the values of the environmental variables for the sampling sites with the help of the tool ‘Sample Raster Values’. The majority of environmental variables followed the same World Geodetic System 84 (WGS84) coordinate reference system. Where this was not the case, layers were reprojected to ensure spatial consistency. We calculated the mean from the monthly solar radiation values to get an annual average. The soil Mg data were obtained in vector format; therefore, the values for the samples were extracted by the tool ‘Join Attributes by Location’. In addition, a spatial index was added, and the geometries were fixed for each vector layer in Qgis (v.3.22). All accessions with missing data were removed from the analysis, resulting in a dataset of 578 accessions.

We first examined the relationship between variation in leaf Mg content and each of the soil and climatic variables using the spearmanr function provided in SciPy (v.1.6.2) (Virtanen *et al*., 2020). We then applied a random forest regression to identify the environmental variables with the most predictive power for leaf Mg variation. For this analysis, we first performed a principal component analysis from genome‐wide single‐nucleotide polymorphism (SNP) data using Plink (v.1.90b6.26) (Purcell *et al*., 2007) and included the first 20 principal components into the model to account for population structure (Table S2). Then, we used 80% of the data for training and the remaining data portion for testing. We conducted a fourfold cross‐validation procedure on the training set to generate the final model using the gridsearchCV and RandomForestRegressor functions in sci‐kit learn (v.0.24.1) (Pedregosa *et al*., 2011). We estimated the coefficient of determination (*R* ^2^) of the final model on the testing set with the r2_score function in scikit‐learn (v.0.24.1) (Pedregosa *et al*., 2011). The most important variables were assessed with the feature_importance function in scikit‐learn (v.0.24.1) (Pedregosa *et al*., 2011) using 1000 permutations.

### Trait mapping

To identify genetic loci associated with natural variation in leaf Mg, we conducted genome‐wide association studies (GWAS) in the Eurasian panel (*n* = 690 accessions) and natural populations from Santo Antão, CVI (*n* = 166). For the Eurasian population, we used the variant call format (VCF) file provided by the 1001 Genomes consortium (The 1001 Genomes Consortium, 2016). For the Santo Antão population analysis, we used the VCF published in Fulgione *et al*. (2022). To ensure high‐quality SNP calls, we filtered VCFs using VCFtools (Danecek *et al*., 2011) to include only bi‐allelic SNPs and variants with coverage above three and base quality above 25. We conducted GWAS using Gemma (v.0.94) (Zhou *et al*., 2013). We retained only variants with minor allele frequency above 5% and missingness below 10%. We generated a centered kinship matrix using ‐gk 1 in Genome‐wide Efficient Mixed Model Association (GEMMA) to account for population structure in the GWAS model.

To characterize the genetic architecture of leaf Mg variation, we calculated the broad‐sense heritability (*H* ^2^) with the following equation:

$$H^{2}=\frac{{\text{variance}}_{\text{line}}}{{\text{variance}}_{\text{line}}+{\text{variance}}_{\text{residual}}}$$

using a linear mixed model (LMM) that accounts for block effects with the lme4 package (v.1.1‐35.5) (Bates *et al*., 2015) in R (v.4.4.2) (R Development Core Team, 2017). We calculated the narrow‐sense heritability (chip heritability) using a Bayesian Sparse Linear Mixed Model approach, which assumes an additive model (Gemma, v.0.94) (Zhou *et al*., 2013). For this, we ran Markov chain Monte Carlo with 10 000 000 sampling steps and 2500 000 burn‐in iterations and calculated the median and 95% confidence interval for the proportion of variance in phenotypes explained by available genotypes (PVE) and the number of large effect loci (n_gamma) across 10 runs. To characterize the genetic basis of leaf Mg variation, we used an LMM approach provided in GEMMA using ‐lmm 4 (v.0.94) (Zhou & Stephens, 2012). We estimated the effect size of a variant using beta, which corresponds to the unit change of the phenotype per copy of the minor allele.

We conducted genomic control using the genomic inflation factor, λ, to assess excess of significant *P*‐values, with SciPy (v.1.6.2) using the following equation:

$$\lambda =\frac{O\left(\chi_{\text{median}}^{2}\right)}{E\left(\chi_{\text{median}}^{2}\right)}$$

where λ represents the genomic control, $O\left(\chi_{\text{median}}^{2}\right)$ corresponds to the observed median of the chi‐squared values, and $E\left(\chi_{\text{median}}^{2}\right)$ corresponds to the expected median of a chi‐squared distribution with one degree of freedom (0.4549).

Pairwise linkage disequilibrium (*r* ^2^) was estimated with PLINK (v.1.90b6.26) (Purcell *et al*., 2007) using ‐‐ld‐window‐kb 1000, ‐‐ld‐window 99 999, ‐‐ld‐window‐r2 0. Linkage disequilibrium (LD) heatmaps were generated using LDBlockShow (Dong *et al*., 2021).

To assess the contribution of the GWAS peaks to the phenotypic variance, we included the SNPs identified through GWAS as covariates in the LMM. For the Eurasian panel, the PVE for all markers was at 58.6%. Once we included the three markers identified at the *RAPTOR1A* and *MGR5* regions as covariates, the percentage of phenotypic variance explained by all remaining markers decreased to 47.3%, indicating that the GWAS peaks accounted for 11.3% of the phenotypic variance. Similarly, for the Santo Antão panel, the PVE for all markers was at 64.9% and decreased to 38.5% once we included the *MGR5 L77fs* variant as a covariate, indicating that the GWAS peak accounted for 26.4% of the phenotypic variance.

To examine the association between the *S470fs MGR5* variant and leaf Mg content variation, we recalled the variant as only the alternative allele has been called in the original VCF from the 1001 Genomes panel. To do this, we first mapped the reads to the reference genome (The Arabidopsis Information Resource (TAIR)10) using the bwa mem algorithm (v.0.7.15) (Li & Durbin, 2009). We then used Picard (v.2.21.1) to mark and sort duplicated reads, and Gatk HaplotypeCaller (v.4.2.3.0) to call indels. The resulting gVCFs were merged using the Genome Analysis Toolkit (GATK) GenotypeGVCFs function (v.4.2.3.0) (Van der Auwera & O'Connor, 2020). The pipeline can be found at https://github.com/HancockLab/SNP_and_Indel_calling_Arabidopsis_GATK4. The resulting VCF was then filtered using the same filters we previously used (Genotype Quality (GQ) > 25, Read Depth (DP) > 3) (Table S3). We then included the *S470fs MGR5* variant in the original VCF and re‐ran GWAS analyses.

To infer ancestral and derived states at each locus, we used variation data from previously sequenced Moroccan samples (Durvasula *et al*., 2017), as Moroccan *A. thaliana* has been found to represent ancient variation in the species. An allelic state was considered ancestral if it was fixed in Morocco. Using this approach, ancestral state assignment would not be possible for variants that segregate in Morocco; however, none of the variants we examined here segregated in Morocco (Table S4).

To examine the relationship between GWAS‐identified variants and latitude, we performed a logistic regression analysis. In this analysis, the presence or absence of the variant was treated as a response variable and latitude as an independent variable. The logistic regression was implemented in R (v.4.4.2) (R Development Core Team, 2017) using the glm() function with a binomial distribution.

To further explore how the GWAS‐identified variants contribute to the observed latitudinal cline in leaf Mg, we built linear regression models in R using the lm() function. In this analysis, latitude was treated as a response variable, and leaf Mg content as an independent variable, with or without accounting for the presence or absence of the variants. To determine the contribution of each variant to the models, we added each variant sequentially and calculated the likelihood ratio using the anova() function in R (v.4.4.2) (R Development Core Team, 2017) with the test set to ‘likelihood ratio test (LRT)’ to compare the models.

### CRISPR mutagenesis

To validate the function of the frameshift variant identified in Santo Antão, we used Clustered Regularly Interspaced Short Palindromic Repeats (CRISPR) to generate *MGR5* frameshift variants in the most relevant background. We used S1‐1 and S15‐3, which have the *MGR5* ancestral allele, and Col‐0 as a control. The CRISPR lines were generated following the method described by Wang *et al*. (2015). Briefly, we identified target sites with the web tool (https://www.genome.arizona.edu/crispr/CRISPRsearch.html). We used oligonucleotides P1–P8 (Table S5) containing the first 19 nucleotides of the target sites in a PCR reaction for insertion into the pCBC‐DT1T2 module vector. The resulting PCR product was introduced into the pHEE401E binary vector via Golden Gate cloning. Stable transgenic bacterial colonies were selected with kanamycin (50 μg ml^−1^) and confirmed by colony PCR using the primers P9 and P10 (Table S5). We then introduced the resulting binary vectors into *Agrobacterium tumefaciens* strain GV3101‐pSOUP by electroporation. We selected stable transgenic bacteria after treatment with rifampicin (50 μg ml^−1^), gentamycin (50 μg ml^−1^), tetracyclin (5 μg ml^−1^), and kanamycin (50 μg ml^−1^). Before plant transformation, stable bacteria were grown to an OD_600_ of 0.7–1.0. The growth media was supplemented with 5% sucrose and Silvet L‐77 (500 μl l^−1^). We transformed the three different accessions (Col‐0, S1‐1, and S15‐3) with the binary vector using the floral dip method (Clough & Bent, 1998). T1 transgenic plants were selected on Murashige and Skoog (MS) plates with hygromycin B (15 μg ml^−1^). T2 plants lacking the Cas9 transgene were identified by PCR using the primers P11 and P12 (Table S5). We Sanger sequenced the edited genomic region by Sanger sequencing using the primers P13–P16 (Table S5). We identified frameshift mutations in each of the three backgrounds, resulting in nonfunctional *MGR5* proteins. In S1‐1‐*mgr5*, we identified a 68‐bp deletion in the first exon of *MGR5*, and in S15‐3‐*mgr5*, we identified a 1‐bp insertion in the second exon of *MGR5*. In our Col‐0‐*mgr5* mutant, we identified a 147‐bp deletion spanning the first and second exons of *MGR5*. The deletion removed 24 bp in the first exon and 32 bp in the second exon, leading to a fusion of the first two exons and a frameshift.

To investigate the potential for off‐target mutations, we generated high‐coverage short‐read genomic data for the CRISPR lines (ranging from 59.7 to 81.1×x). DNA was extracted with the DNeasy Plant Mini Kit (Qiagen, catalog no. 69106). The libraries were prepared using NEBNext Ultra II FS DNA Library Prep (Illumina, New England Biolabs (NEB), Ipswich, MA, USA). Sequencing was conducted on an Illumina HiSeq3000 platform (Illumina, NEB) with 150‐bp paired‐end reads. The resulting reads were mapped to TAIR10 with bwa (v.0.7.15) using the mem algorithm. We used GATK to call SNPs and short indels as described in Tergemina *et al*. (2022) (https://github.com/HancockLab/Fogo‐Edaphic/tree/main/Supplementary/FigS12). We used Cas‐OFFinder (Bae *et al*., 2014) to predict potential off‐target sites on the TAIR10 reference genome, allowing a maximum of five mismatches in the target sites. We used SpCas9 from *Streptococcus pyogenes* (5′‐NGG‐3′) as CRISPR‐Cas–derived RNA‐guided endonuclease. Finally, we examined evidence for overlap between predicted off‐target sites and SNPs and indels with a custom Python script (https://github.com/HancockLab/Fogo‐Edaphic). We found no evidence of off‐target mutations in the CRISPR lines. To compare results from GWAS and CRISPR lines, we reported variant effects as two times the beta from the GWAS.

## Results

**Fig. 1:**
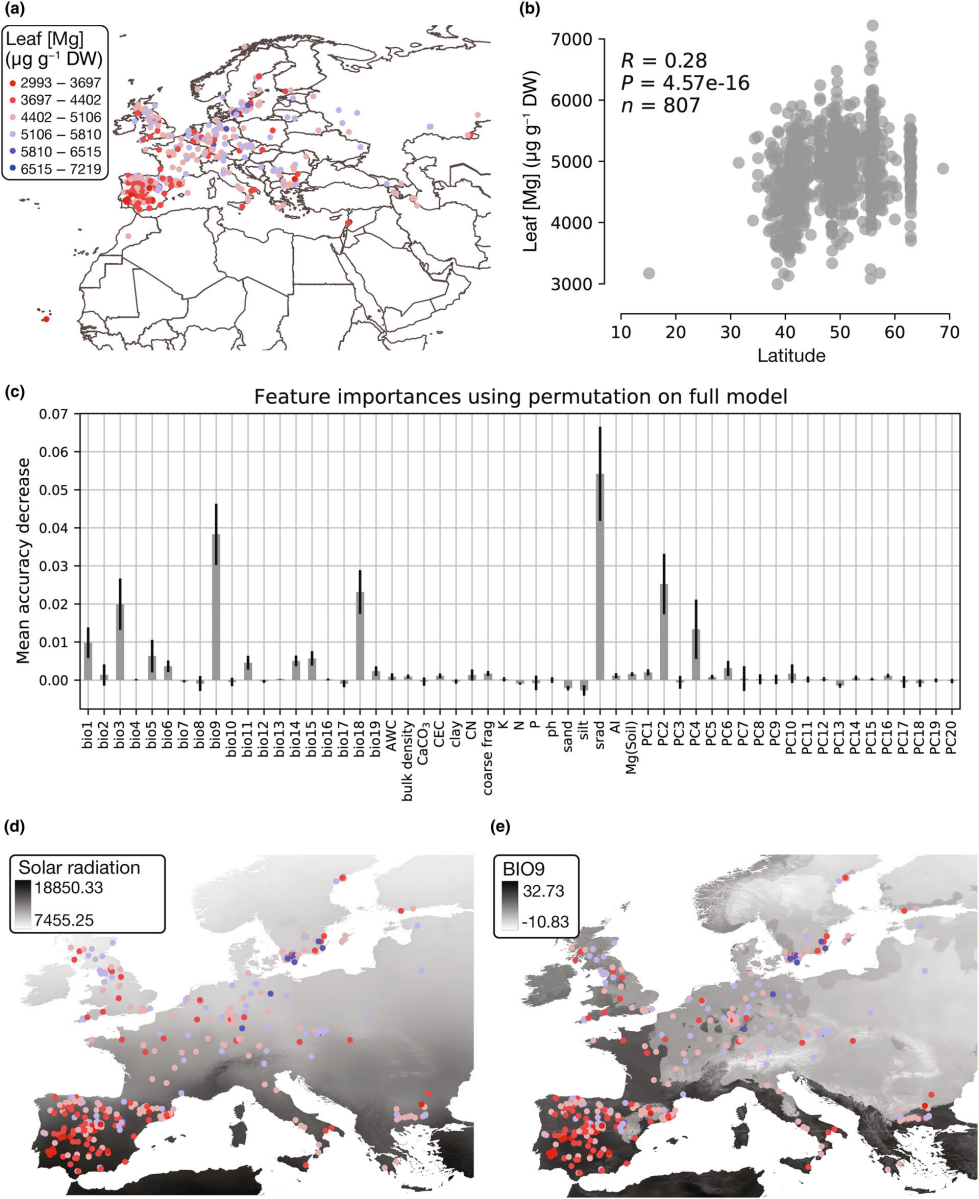
Environmental factors influencing leaf magnesium (Mg) content in *Arabidopsis thaliana*. (a) Sample map of accessions included in the global panel (for an overview of the accessions, see Supporting Information Table S1). Each circle represents a plant at its latitude and longitude of origin. The color indicates leaf Mg content in μg g^−1^ of dry weight. (b) Correlation between leaf Mg content and latitude of origin. *R* = Spearman's rho, *P* = *P* value, *n* = number of accessions. (c), Feature importance using permutation on a random forest regression. The *x*‐axis corresponds to the variables included in the model. The *y*‐axis corresponds to the mean decrease in accuracy when the variable is excluded from the model. Error bars correspond to SD. srad, solar radiation; AI, aridity index; AWC, available water capacity; CN, carbon nitrogen ratio; CEC, cation exchange capacity. PC1–PC20 correspond to the first 20 principal components of a matrix of genome‐wide SNPs. (d) and (e) Maps of the two most important predictive variables. Values are in kJ m^−2^ d^−1^ for solar radiation and in °C for the mean temperature of the driest quarter (BIO9). The color indicates leaf Mg content with the same scale as in Fig. 1.

We investigated the factors that impact Mg accumulation across a global set of *A. thaliana* accessions. For this, we used leaf Mg content assayed from plants grown under common experimental conditions (Campos *et al*., 2021) so that observed variation among accessions reflects genetic differences in Mg accumulation. We found that Mg accumulation tended to be highest in leaves from high latitude populations, including those from Sweden (Liarum (8241), TV‐38 (6284), TDr‐18 (6203), St‐0 (8387), and Lan‐1 (9421)) and lowest in lower latitude populations in Spain (IP‐ReI‐0 (9574), IP‐Ala‐0 (9515), IP‐Lam‐0 (9855)), and the CVI (Cvi‐0 (6911)) (Fig. 1a; Table S1). Overall, leaf Mg accumulation was significantly correlated with latitude (Spearman's rho = 0.33, *P* = 1.59 × 10^−16^) (Fig. 1b). To identify the ecological drivers underlying the latitudinal cline in Mg we observed in *A. thaliana* leaves, we focused on the Eurasian population where most of the accessions from the 1001 Genomes panel originate. Recent research indicates that soils in southern Europe have more Mg than those in northern Europe primarily due to the parental material and chemical weathering (Négrel *et al*., 2021). We used 14 soil variables (Ballabio *et al*., 2019; Négrel *et al*., 2021) and 21 environmental variables (Fick & Hijmans, 2017) at collection sites to perform a random forest regression that accounts for population structure (Table S1). Our model explained 30% of the variation in leaf Mg (*R* ^2^ = 0.30), revealing that the variables with the most predictive power were solar radiation and the mean temperature of the driest quarter (BIO9) (Fig. 1c), followed by precipitation during the driest quarter (BIO18) and isothermality (BIO3). Specifically, accessions with higher leaf Mg tended to come from geographic regions with lower solar radiation (Spearman's rho = −0.33, *P* = 1.57 × 10^−16^), lower temperatures during the driest quarter (Spearman's rho = −0.38, *P* = 8.27 × 10^−21^), higher precipitation during the warmest quarter (Spearman's rho = 0.33, *P* = 1.68 × 10^−16^), and lower isothermality (Spearman's rho = −0.36, *P* = 1.69 × 10^−19^) (Figs 1d,e, S1–S4). By contrast, soil variables at collection sites had only weak predictive power. Overall, we find a latitudinal cline in the spatial distribution of Mg accumulation in *A. thaliana* and identify climatic factors, including solar radiation, temperature, and precipitation, that may contribute to variation in Mg homeostasis across the landscape.

**Fig. 2:**
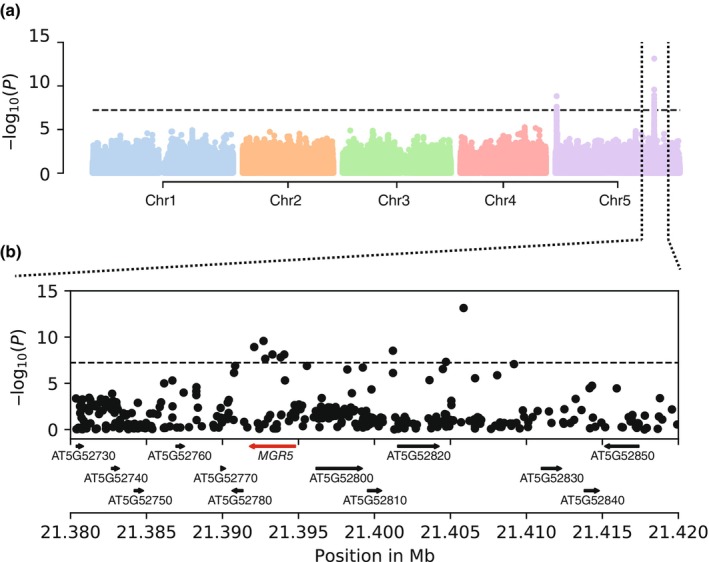
MAGNESIUM RELEASE 5 (*MGR5*) is associated with leaf magnesium (Mg) variation in *Arabidopsis thaliana*. (a) Genome‐wide association studies for leaf Mg variation in the Eurasian population (690 accessions). The dashed horizontal line corresponds to the 5% Bonferroni‐adjusted genome‐wide significance threshold. (b) Magnification of the peak at the *MGR5* region. The dashed horizontal line corresponds to the 5% Bonferroni‐adjusted genome‐wide significance threshold. *MGR5* is highlighted in red.

To characterize the genetic architecture of leaf Mg accumulation in a diversity panel of Eurasian *A. thaliana* accessions, we used leaf Mg estimates from Campos *et al*. (2021) that overlapped with the 1001 Genome panel (*n* = 690 accessions) (The 1001 Genomes Consortium, 2016; Campos *et al*., 2021). We estimated broad‐sense heritability (*H* ^2^) based on repeatability across replicates and narrow‐sense heritability based on the proportion of phenotypic variance explained by available genotypes (PVE) (Zhou *et al*., 2013). We found that a moderate proportion of the variation in leaf Mg could be explained by genetic factors (*H* ^2^ = 28.3%, PVE median = 60.4%, 95% confidence interval (CI) = 49.7–70.3), with contributions from an estimated nine loci (median estimate = 9, 95% CI = 3–34). To map the genetic basis of leaf Mg variation, we conducted GWAS using an LMM approach that controls for population structure through the inclusion of a relatedness matrix (Zhou & Stephens, 2012). We identified two Bonferroni significant peaks on chromosome 5: one at the top of the chromosome and the other *c*. 5.5 Mb from the bottom of the chromosome (Fig. 2a).

The peak at the top of chromosome 5 contained no candidates previously implicated in Mg homeostasis (Fig. S5; Table S6). The two most significant SNPs lie *c*. 1 kb downstream of AT5G01740, an uncharacterized *NUCLEAR TRANSPORT FACTOR 2 family protein* gene highly expressed in root tissues. Candidate genes in the region include AT5G01730, a gene encoding a member of the suppressor of cAMP receptor/Wiskott‐Aldrich syndrome protein‐family verprolin‐homologous protein (SCAR/WAVE) complex, which regulates filamentous actin nucleation (Zhang *et al*., 2008); miR164b (AT5G01747), a microRNA involved in a broad range of processes, including flower, leaf, and lateral root development (Baker *et al*., 2005; Hs *et al*., 2005; Nikovics *et al*., 2006); and *REGULATORY‐ASSOCIATED PROTEIN OF TOR 1A* (*RAPTOR1A*, AT5G01770). *RAPTOR1A* represents a promising candidate because it encodes a member of the target of rapamycin complex 1 (TORC1), which affects ATP levels in plant cells and coordinates cell growth and metabolism in response to nutrients (Dobrenel *et al*., 2016; Dai *et al*., 2022; Cho *et al*., 2023; Li *et al*., 2023). When we added the most significant SNP (chr5:282011, T/C) in this peak to the GWAS model, no significant variants remained in the region, implying that a single haplotype can explain the signal (Fig. S5). Accessions carrying the derived allele at chr5:282011 accumulate more Mg in leaf tissues (beta = 367.3 μg g^−1^ of dry weight). This derived allele segregates at an intermediate frequency (*c*. 18.3%) in our dataset, with a broad geographic distribution across Eurasia (logistic regression: *P* = 0.59) (Fig. S5). Taken together, our results suggest that a mutation coincident with the *RAPTOR1A* region contributes to leaf Mg variation *in A. thaliana*, increasing leaf Mg content in the derived state.

**Fig. 3:**
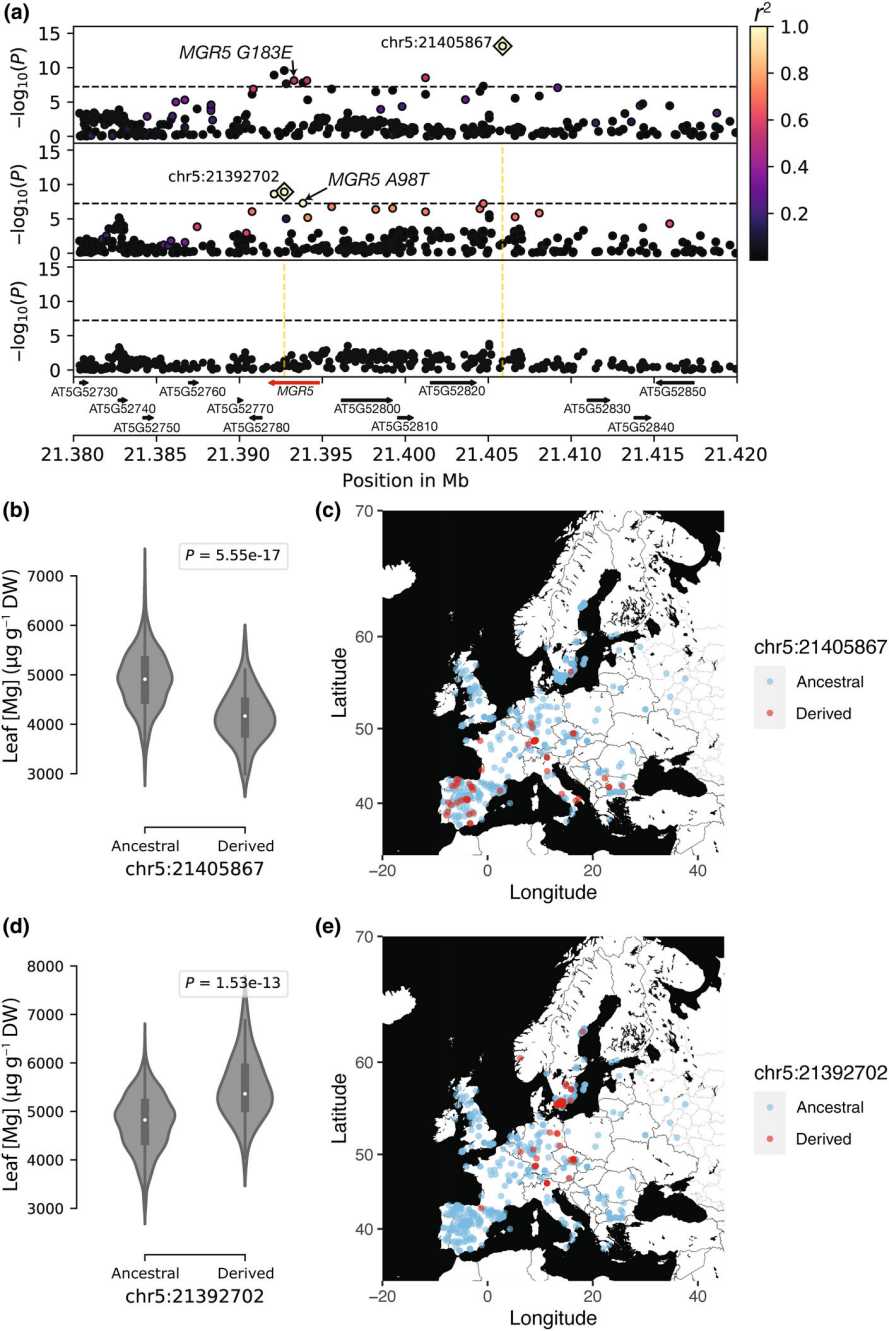
Allelic heterogeneity at MAGNESIUM RELEASE 5 (*MGR5*) contributes to leaf magnesium (Mg) variation in *Arabidopsis thaliana*. (a) Magnification of the peak at the *MGR5* region. The top panel corresponds to the unconditioned genome‐wide association studies (GWAS). The middle panel corresponds to the GWAS after conditioning for chr5:21405867. The bottom panel corresponds to the GWAS after conditioning for chr5:21405867 and chr5:21392702. Dashed horizontal lines correspond to the 5% Bonferroni‐adjusted genome‐wide significance threshold. Colors indicate linkage disequilibrium to the focal SNP (*r* ^2^) annotated with a diamond. *MGR5* is highlighted in red. The difference in leaf Mg content in μg g^−1^ of dry weight between the *MGR5* chr5:21405867 (b) and the *MGR5* chr5:21392702 alleles (d). White dots of violin plots represent the median, boxes denote the 25^th^ and 75^th^ percentiles, and whiskers extend from the box to the farthest data point lying within 1.5 times the inter‐quartile range from the box. *P* = *P* value for Mann–Whitney–Wilcoxon (MWW) test. Geographic distribution of the *MGR5* chr5:21405867 (c) and the *MGR5* chr5:21392702 (e) alleles in Europe.

The peak at the bottom of chromosome 5 mapped to *MGR5* (AT5G52790) (Fig. 2b). *MGR5* is a strong candidate because it encodes a plasma membrane Mg transporter involved in root‐to‐shoot Mg partitioning (Meng *et al*., 2022; Tang *et al*., 2022). Among the nine SNPs in the *MGR5* region that were significantly associated with leaf Mg variation, five were located within *MGR5* (Fig. 3a). The most significant SNP (chr5:21405867, A/G), located *c*. 11 kb downstream of *MGR5*, showed among the highest LD (*r* ^2^ = 0.51) with a missense variant at *MGR5* (chr5:21393289, *G183E*), but was in poor LD with the other SNPs at *MGR5*, suggesting that multiple alleles at the locus may contribute to variation in leaf Mg content. To investigate this further, we performed a stepwise conditional GWAS and found that at least two independent SNPs were needed to explain the signal in the *MGR5* region, which could be represented by the top SNP at the *MGR5* peak (chr5:21405867) and the second most significant SNP, which is a synonymous variant at *MGR5* (chr5:21392702, G/C). The second most significant SNP is in perfect LD (*r* ^2^ = 1) with a missense variant at *MGR5* (chr5:21393829, *A98T*) 1.1 kb downstream and in partial LD (*r* ^2^ = 0.75) with a frameshift variant at *MGR5* (chr5:21391927, *S470fs*) (Figs 3a, S6, S7). The two *MGR5* haplotypes segregate in our dataset at moderate frequencies (chr5:21405867 = 8.8%, chr5:21392702 = 8.1%, Table S6), and the derived alleles show contrasting patterns in their association with Mg accumulation. The derived chr5:21405867 variant is associated with a decrease in leaf Mg content (beta = −603.5 μg g^−1^ of dry weight) and is distributed mainly in southern latitudes (logistic regression: *P* = 2.8 × 10^−7^) (Fig. 3b,c). By contrast, the derived chr5:21392702 variant is associated with an increase in leaf Mg content (beta = 483.9 μg g^−1^ of dry weight after conditioning for chr5:21405867) and is found mainly in northern latitudes (logistic regression: *P* = 8.5 × 10^−5^) (Fig. 3d,e). Furthermore, a linear model indicated that the two *MGR5* variants contributed significantly to the latitudinal cline in leaf Mg content we observed (adjusted *R* ^2^ = 8.4%; chr5:21405867: *β* = −3.7, *P* = 6.21 × 10^−4^; chr5:21392702: *β* = 2.6, *P* = 1.8 × 10^−2^) (Table S7).

When we applied an LMM conditioned for the *RAPTOR1A* region (chr5:282011) and both the *MGR5* (chr5:21392702 and chr5:21405867) alleles, we found that two regions explained 11.3% of the phenotypic variation. Furthermore, no Bonferroni significant peak remains, indicating that the two regions additively contribute to variation and represent the major genetic factors affecting variation in leaf Mg content within the Eurasian panel (Fig. S8). Taken together, the GWAS results suggest that natural variation at *MGR5* contributes to the latitudinal cline in leaf Mg accumulation.

Identifying causative variants in continental populations can be challenging due to complex genetic structure and allelic heterogeneity. By contrast, local populations offer an excellent alternative, as they may lack these complexities (Gloss *et al*., 2023). Of the accessions included in Campos *et al*. (2021), Cvi‐0 derives from the lowest latitude (*c*. 16°N) and is also among the samples with the lowest leaf Mg content. This accession is thus both a phenotypic and genetic outlier relative to the Eurasian population. Therefore, including Cvi‐0 in GWAS on the global population was not advisable because this would violate the assumptions of the linear model approach. Further, variants private to CVI would have been filtered from the GWAS because of their low frequencies. However, given the observation that Mg content is particularly low in Cvi‐0, we were interested in probing the CVI population to identify genetic drivers of low Mg there.

Cvi‐0 was originally collected in Santo Antão, a volcanic island in the CVI archipelago *c*. 570 km away from the coast of Senegal (Fulgione *et al*., 2022). To investigate Mg accumulation in Santo Antão, CVI population more generally, we measured leaf Mg content on a panel of 166 *A. thaliana* accessions collected from across the island (Fulgione *et al*., 2022) under the same conditions used by Campos *et al*. (2021). We found that Santo Antão accessions accumulate less Mg in their leaves on average compared to Eurasian accessions (MWW test, *P* = 1.97 × 10^−16^; Fig. S9; Table S8). This is consistent with the results in Cvi‐0 and the overall clinal pattern in the global population set.

**Fig. 4:**
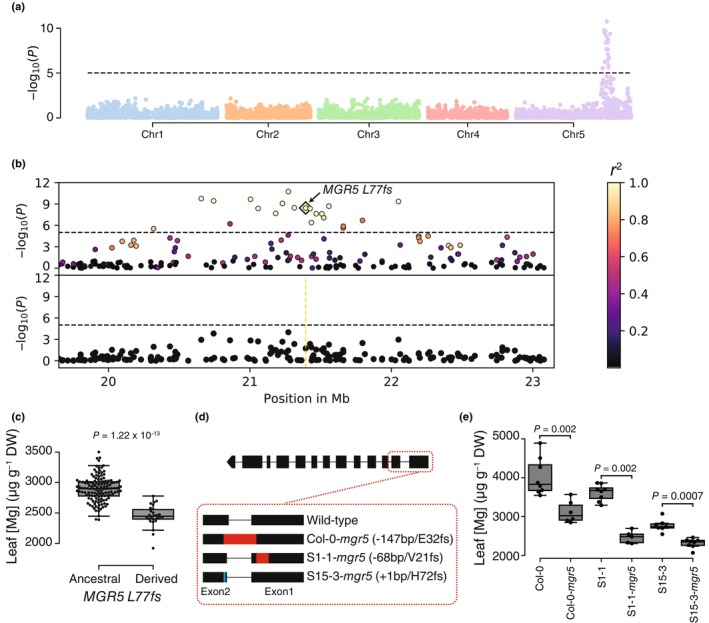
A frameshift variant of MAGNESIUM RELEASE 5 (*MGR5*) reduces leaf magnesium (Mg) in *Arabidopsis thaliana* from Cape Verde. (a) Genome‐wide association studies (GWAS) for leaf Mg variation in the Santo Antão population. (b) Magnification of the peak at the *MGR5* region. The upper panel corresponds to the unconditioned GWAS. The lower panel corresponds to the GWAS after conditioning for *MGR5 L77fs*. Dashed horizontal lines correspond to the 5% Bonferroni‐adjusted genome‐wide significance threshold. Colors indicate linkage disequilibrium to the focal SNP (*r* ^2^) annotated with a diamond. (c) Difference in leaf Mg concentration between the two *MGR5 L77fs* alleles in μg g^−1^ of dry weight (DW). *P* = *P* value for MWW test. (d) Representation of the *MGR5* gene indicating the frameshift mutants generated for this study using the CRISPR‐Cas9 system. (e) *mgr5* mutants show less Mg in the leaves compared to their respective wild‐types. *P* = *P* value for MWW test. Center lines of box plots represent the median, boxes denote the 25^th^ and 75^th^ percentiles, whiskers extend from the box to the farthest data point lying within 1.5 times the interquartile range from the box, and dots represent individual data points.

Heritability of Mg accumulation in the Santo Antão population from CVI was high (*H* ^2^ = 52%, PVE median = 65.2%, 95% CI = 53.5–75), with fewer large‐effect loci (median estimate = 2, 95% CI = 1–11) contributing to the trait variation compared to the Eurasian panel (median estimate = 9, 95% CI = 3–34). As in the Eurasian panel, GWAS for leaf Mg content revealed a major peak on chromosome 5, corresponding to *MGR5* (Fig. 4a). The segregating variation in CVI is distinct from that in mainland populations due to the extreme bottleneck and subsequent isolation after the founding of the island populations (Fulgione *et al*., 2022), implying that the observed signal is driven by an allele independent of those found in the 1001 Genomes panel. Within the CVI peak region, we identified a five‐bp deletion (chr5:21394005–21394009) in the first exon of *MGR5* leading to a frameshift variant (*L77fs*) and resulting in a nonfunctional protein (Table S9). This mutation is unique to the Santo Antão population, where it segregates at *c*. 23% frequency (Table S10). The peak at *MGR5* disappeared when we conditioned for the *MGR5 77fs* variant, indicating that the variant is sufficient to explain the signal at the *MGR5* region (Figs 4b, S10). The *MGR5 77fs* variant explained 26.4% of the phenotypic variation and, as expected from a loss of MGR5 function, was associated with a decrease in leaf Mg content in the Santo Antão population (beta = −419.6 μg g^−1^ of dry weight) (Fig. 4c). Since the variant is not found in the Eurasian panel, it cannot be responsible for the patterns we observed there and thus represents a novel allele relative to the world‐wide distribution of *A. thaliana*. Overall, our results strongly suggest that the major effect conferred on the protein by this frameshift can explain the variation in leaf Mg content associated with the GWAS peak in Santo Antão.

Next, we tested for a functional effect of MGR5 disruption in the CVI and reference strain (Col‐0) backgrounds using gene editing. We used the CRISPR‐Cas9 system to generate null mutants of *MGR5* in two accessions from Santo Antão: S1‐1 (S1‐1*‐mgr5*) and S15‐3 (S15‐3‐*mgr5*), which both carried the ancestral *MGR5* allele, as well as in the reference accession Col‐0 (Col‐0*‐mgr5*) (Figs 4d, S11). Consistent with our GWAS results and previous studies of *MGR5* (Meng *et al*., 2022), *mgr5* CRISPR mutants had lower leaf Mg than the corresponding wild‐type accessions (Fig. 4e). Interestingly, the effect of the CRISPR‐induced loss of MGR5 was stronger than that expected based on GWAS results in the Santo Antão population (beta = −419.6 μg g^−1^ of dry weight, Col‐0‐*mgr5* = −808 μg g^−1^ of dry weight, S1‐1‐*mgr5* = −1190 μg g^−1^ of dry weight, S15‐3‐*mgr5* = −388 μg g^−1^ of dry weight). Since effect size estimates from GWAS are averaged across genetic backgrounds within the population, they can be impacted by variation in other genetic factors and population structure. Therefore, comparing the statistical estimates of effect size in a population to those measured in mutant lines in different genetic backgrounds provides a clearer view of the actual impact of the gene disruption itself. Together, our results demonstrate that functional variants in *MGR5* contribute to leaf Mg variation in *A. thaliana* and that multiple independent *MGR5* variants have arisen across the world‐wide distribution of *A. thaliana*.

## Discussion

Understanding the genetic and environmental determinants of nutrient accumulation in plants is critical for tailoring crops to local environments and predicting crop responses to climate change. Despite numerous studies in nutrient homeostasis, the factors influencing Mg accumulation in widespread plant species remain limited. Here, we studied a widespread Eurasian population of *A. thaliana* as well as a local CVI population to explore potential factors influencing leaf Mg accumulation across the species range. Since the CVI population contains distinct genetic variation compared to the mainland and is both a phenotypic and genetic outlier relative to Eurasia, it was necessary to conduct analyses separately for these two populations.

### The genetic basis of leaf Mg acquisition in *A. thaliana*

We examined the genetic basis of variation in leaf Mg accumulation using a GWAS approach. Our results indicated that variation in leaf Mg content within Eurasian populations is moderately polygenic, with substantial contributions from two loci at the *RAPTOR1A* and *MGR5* regions.

*RAPTOR1A* stood out due to its involvement in the TORC1 complex, which integrates environmental factors and nutrient sensing to regulate plant growth (Dobrenel *et al*., 2016; Dai *et al*., 2022; Cho *et al*., 2023). In particular, recent studies highlighted the cross‐talk between TORC1 and the calcineurin B‐like protein (CBL)–CBL‐interacting kinase regulatory pathway, which contributes to Mg homeostasis (Tang *et al*., 2015; Li *et al*., 2023). Together, our results warrant further investigation into the role of RAPTOR1A in the TORC1 complex and its broader impact on Mg homeostasis.

At *MGR5*, we found evidence that multiple haplotypes contribute to variation in leaf Mg accumulation in Eurasia; our GWAS results indicated that at least two *MGR5* haplotypes are required to explain the observed signal in Eurasia.

In addition to the Eurasian population, we also investigated the genetic factors that contribute to variation in a local population sample from CVI. By combining mapping in a widespread Eurasian population with a complex demographic history and an isolated island population with a short history (*c*. 5000 yr), where trait architecture is expected to be less complex (Neto & Hancock, 2023), we were able to better understand global trait evolution and the functional impacts of *MGR5* variation. First, we showed that *MGR5* variation that impacts Mg accumulation has arisen independently across disparate geographic regions. Second, in CVI, we indeed found a clear association at *MGR5* that could be explained by a single variant (*MGR5 77fs*) that was private to the island and predicted to disrupt *MGR5* function. These results reveal a clear case of allelic heterogeneity by demonstrating that multiple *MGR5* alleles contribute substantially to the variation in leaf Mg accumulation across the species range.

*MGR5* is an excellent candidate for variation in Mg accumulation because mutations at *CNNM2*, a human homolog of *MGR*, have been linked to hypomagnesemia (Stuiver *et al*., 2011, p. 2; García‐Castaño *et al*., 2020, p. 2). Furthermore, recent studies indicated that MGR5 belongs to the clade II MGRs, which consist of four MGR transporters (MGR4, MGR5, MGR6, and MGR7) contributing to the translocation of Mg from the root to the shoot. We validated the effect of the disruption of MGR5 in the Santo Antão genetic background using CRISPR in Santo Antão lines. Consistent with previous studies (e.g. Meng *et al*., 2022), we found that the disruption of MGR5 reduced leaf Mg accumulation and explained the signal at *MGR5*. Furthermore, the effects measured in the CRISPR gene‐edited mutants indicated that the statistical effect size estimate from the GWAS was likely an underestimate. This suggests that there may be antagonistic effects operating in the natural population or insufficient power to accurately estimate the effect size there and highlights the benefits of conducting CRISPR mutagenesis in the most relevant genetic background.

### Environmental factors associated with leaf Mg content variation

Latitudinal clines within Eurasian *A. thaliana* populations have been reported for various traits, including flowering time, freezing tolerance, and seed dormancy (Stinchcombe *et al*., 2004; Zhen & Ungerer, 2008; Debieu *et al*., 2013). These latitudinal clines are largely shaped by environmental factors correlated with latitude, such as photoperiod and temperature. Clines can also result from opposing patterns of selection and genetic trade‐offs (Ågren *et al*., 2013; Lee *et al*., 2024).

Our initial analysis of leaf Mg variation in the 1001 Genomes set revealed a latitudinal cline in the spatial distribution of the trait. Given that Mg availability is influenced by edaphic and environmental factors, and that its deficiency limits agriculture, we investigated the factors contributing to the latitudinal gradient in leaf Mg. Using a machine learning approach, we identified several lines of research for potential future investigation. First, solar radiation emerged as a major contributor to the latitudinal cline in Mg accumulation. Mg is a key structural component of Chl, a necessary molecule for energy balance in plants (Cakmak & Yazİcİ, 2010). Thus, our results suggest that variation in leaf Mg may reflect a need to adjust photosynthetic activity in response to solar radiation. Second, the temperature during the driest quarter appeared to be a major factor for the latitudinal cline in Mg accumulation. This bioclimatic variable distinguishes temperate Mediterranean climates, characterized by dry summers, and cold climates, characterized by dry winters, thus contrasting southern and northern Europe (Peel *et al*., 2007). Specifically, plants originating from temperate Mediterranean climates (southern Europe) accumulate less Mg in leaf tissue compared to plants originating from cold climates (northern Europe), consistent with the latitudinal cline we observed. Third, we found that plants that accumulated more Mg in leaf tissues tended to originate from locations with higher precipitation during the warmest quarter. Given that Mg is highly susceptible to leaching (Grzebisz, 2011; Gransee & Führs, 2013), a plausible hypothesis is that the strong predictive power of this variable reflects the high mobility of Mg in soils.

Somewhat surprisingly, we found that soil variables at collection sites had minimal predictive power for leaf Mg content, despite the difference in soil composition between southern and northern Europe. In particular, one would expect soil Mg to be among the strongest predictors, given that southern European soils contain more Mg than those in northern Europe (Négrel *et al*., 2021). However, the relatively coarse resolution of the soil Mg data, compared to other variables in our study, may limit power for its detection as a factor driving Mg accumulation. Overall, our results align with previous observations indicating that climatic variables may drive spatial variation in the nutrient compositions of plants (Reich & Oleksyn, 2004; Han *et al*., 2011; Baxter & Dilkes, 2012; Sun *et al*., 2013).

We also examined the *MGR5* haplotype distributions in the context of the latitudinal cline in Mg. We found the cline could be partially explained by the *MGR5* haplotypes, which showed opposing patterns in their geographic distributions and effects on leaf Mg accumulation. The *MGR5* haplotypes associated with an increase in leaf Mg accumulation are predominant in northern latitudes, while the *MGR5* haplotypes linked to a decrease in leaf Mg accumulation are predominant in southern latitudes. These results suggest that natural variation at *MGR5* contributes to the latitudinal cline in leaf Mg accumulation we observed. Further, our findings support previous modeling, showing that patterns at multiple loci or alleles can drive trait associations with environmental variation (Lotterhos, 2023).

There are still many open questions about the role climate plays in determining nutrient requirements and their acquisition in nature as well as potential ecological trade‐offs that might contribute to the clinal pattern in Mg accumulation. Here, we took an initial step toward this goal by identifying environmental variables that may influence plant Mg acquisition. Future work will be important to clarify the mechanisms that link climatic factors to nutrient status.

### Conclusion

Understanding the genetic and environmental factors determining nutrient accumulation in plants is essential for developing crops optimized for local environments and predicting how crops will respond to climate change. Together, our results reveal a clinal pattern in the spatial distribution of leaf Mg accumulation in widespread plant species. We provide new insights into the genetic basis of Mg accumulation with two major regions: *RAPTOR1A* and *MGR5*. Further, the pattern at *MGR5* informs us about the genetic basis of the latitudinal cline in leaf Mg accumulation we observed. We also show that climate can explain a substantial variation in Mg accumulation and potentially drive this latitudinal cline. Overall, our results indicate that the latitudinal cline in Mg accumulation in *A. thaliana* is not due to random processes. Instead, genetic differences among accessions appear to optimize Mg homeostasis according to their native environmental conditions. Our findings underscore the need for further research to explore the role of environmental variation in Mg homeostasis and the potential effect of global climate change on nutrient accumulation in plants.

## Competing interests

None declared.

## Author contributions

ET and AMH conceived and designed the study. SA and ET performed the environmental analyses; SA, ET and AMH contributed to its interpretation. DES conducted ionomics analysis and contributed to its interpretation. ET and AMH wrote the paper with inputs from all authors.

## Disclaimer

The New Phytologist Foundation remains neutral with regard to jurisdictional claims in maps and in any institutional affiliations.

## Supporting information


**Fig. S1** Correlation between leaf Mg, soil, and bioclimatic variables.
**Fig. S2** Correlation between leaf Mg and 19 bioclimatic variables.
**Fig. S3** Correlation between leaf Mg and other variables.
**Fig. S4** Maps of the two most important predictive variables after solar radiation and BIO9.
**Fig. S5** One SNP (chr5:282011) explains the *RAPTOR1A* signal in the Eurasian panel.
**Fig. S6** Two independent SNPs explain the *MGR5* signal in the Eurasian panel.
**Fig. S7** Magnification of the peak at the *MGR5* region when including the *MGR5 S470fs* variant.
**Fig. S8** The *RAPTOR1A* and *MGR5* regions explain most of the variation in leaf Mg content in the Eurasian panel.
**Fig. S9** Santo Antão accessions accumulate less Mg in leaf tissues compared to Eurasian accessions.
**Fig. S10** Conditional GWAS for leaf Mg variation in Santo Antão.
**Fig. S11** *MGR5* knock‐out alleles generated in this study.

**Table S1** Accession information with bioclimatic and soil variables.
**Table S2** Principal component analysis using genetic data from 578 European *Arabidopsis thaliana* accessions.
**Table S3** GATK call at *MGR5 S470fs* (chr5:21391927) in the Eurasian panel.
**Table S4** SNP identified through GWAS for leaf Mg variation in the European panel with information for the Moroccan samples.
**Table S5** Primers used in this study.
**Table S6** Genome‐wide significant SNP for leaf Mg variation in the Eurasian panel.
**Table S7** Contribution of the variants identified through GWAS for leaf Mg variation in the European panel to the latitudinal cline in leaf Mg.
**Table S8** Difference in leaf Mg between Europe and Santo Antão.
**Table S9** Genome‐wide significant SNP for leaf Mg variation in Santo Antão.
**Table S10** Genotype information at *MGR5 L77fs* (chr5:21394004) in Santo Antão accessions based on the VCF published in Fulgione *et al*. (2022).
Please note: Wiley is not responsible for the content or functionality of any Supporting Information supplied by the authors. Any queries (other than missing material) should be directed to the *New Phytologist* Central Office.

## Data Availability

Short‐read data generated in this study have been deposited in NCBI SRA under the project number PRJNA1080624. We used previously published sequence data from the European Nucleotide Archive (ENA) project ID PRJNA273563 and mapped the reads to the Arabidopsis TAIR reference assembly GCA_000001735.1. We used previously published genomic variant calls from https://1001genomes.org/data/GMI‐MPI/releases/v3.1/ and from the ENA projects PRJEB44201 and PRJEB24044. Source data and scripts can be found in the GitHub repository (https://github.com/HancockLab/Leaf‐Mg) and on Zenodo (doi: 10.5281/zenodo.11149656).
