# Supplementary material for: Multiple independent MGR5 alleles contribute to a clinal pattern in leaf magnesium across the distribution of Arabidopsis thaliana
Source: New Phytol. 2025 Mar 24;246(4):1861–74. doi: 10.1111/nph.70069 (PMC12018779; doi:10.1111/nph.70069)
Supplement: Supplementary file 1 — Fig. S1 Correlation between leaf Mg, soil, and bioclimatic variables. Fig. S2 Correlation between leaf Mg and 19 bioclimatic variables. Fig. S3 Correlation between leaf Mg and other variables. Fig. S4 Maps of the two most important predictive variables after solar radiation and BIO9. Fig. S5 One SNP (chr5:282011) explains the RAPTOR1A signal in the Eurasian panel. Fig. S6 Two independent SNPs explain the MGR5 signal in the Eurasian panel. Fig. S7 Magnification of the peak at the MGR5 region when including the MGR5 S470fs variant. Fig. S8 The RAPTOR1A and MGR5 regions explain most of the variation in leaf Mg content in the Eurasian panel. Fig. S9 Santo Antão accessions accumulate less Mg in leaf tissues compared to Eurasian accessions. Fig. S10 Conditional GWAS for leaf Mg variation in Santo Antão. Fig. S11 MGR5 knock‐out alleles generated in this study. [file NPH-246-1861-s001.pdf]

New Phytologist

New Phytologist Supporting Information

Article title: Multiple independent *MGR5* alleles contribute to a clinal pattern in leaf magnesium across the distribution of *A. thaliana.*

Authors: Emmanuel Tergemina, Shifa Ansari, David E. Salt and Angela M. Hancock

Article acceptance date: 25 February 2025

The following Supporting Information is available for this article:

Figures S1 to S11

Other supporting materials for this manuscript include the following [see Excel file]:

Table S1 to S10

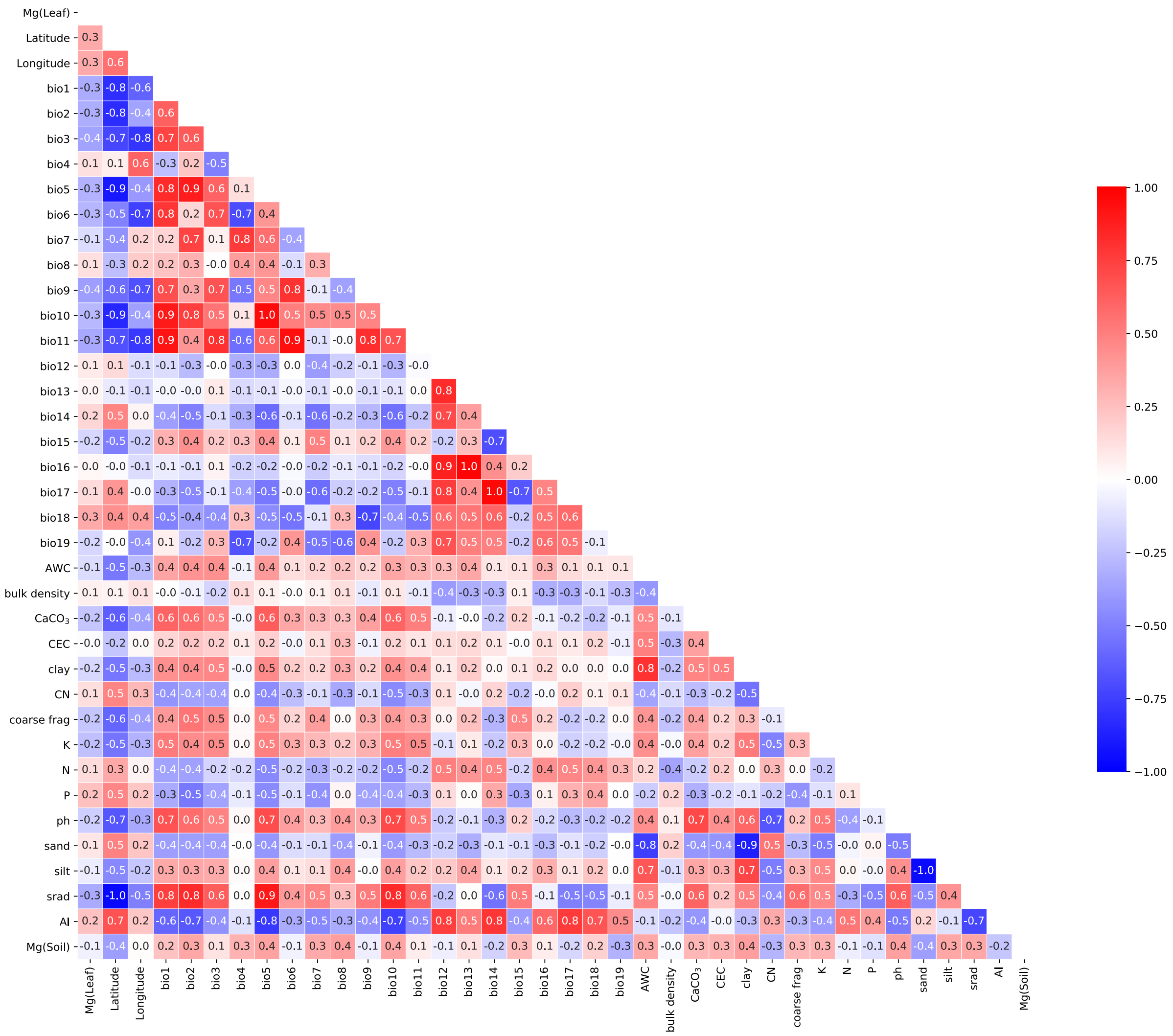

Fig. S1. Correlation between leaf Mg, soil and bioclimatic variables.

Heatmap of the correlations between leaf Mg, soil and bioclimatic variables. Numbers shown in the heatmap correspond to Spearman correlation coefficient. n = 578 accessions. srad = solar radiation, AI = aridity index, AWC = available water capacity, CN = carbon nitrogen ratio, CEC = cation exchange capacity.

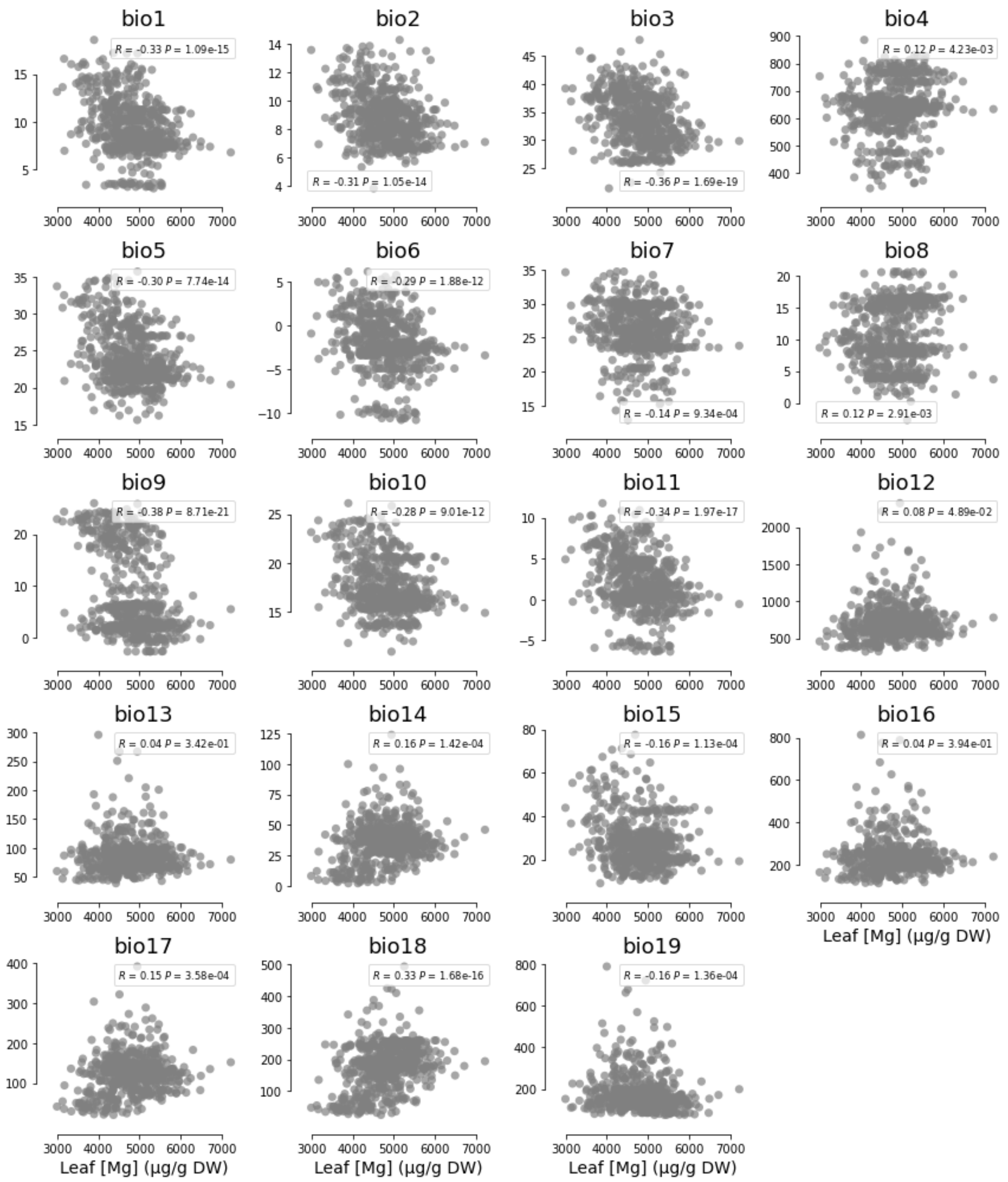

Fig. S2. Correlation between leaf Mg and 19 bioclimatic variables.

Leaf Mg concentrations are shown on the x-axis in µg/g of dry weight (DW). *R* = Spearman's rho, *P* = *P* value, n = 578 accessions, bio1 = annual mean temperature, bio2 = mean diurnal range (mean of monthly (max temp - min temp)), bio3 = isothermality (bio2/bio7) (×100), bio4 = temperature seasonality (standard deviation ×100), bio5 = max temperature of warmest month, bio6 = min temperature of coldest month, bio7 = temperature annual range (bio5-bio6), bio8 = mean temperature of wettest quarter, bio9 = mean temperature of driest quarter, bio10 = mean temperature of warmest quarter, bio11 = mean temperature of coldest quarter, bio12 = annual precipitation, bio13 = precipitation of wettest month, bio14 = precipitation of driest month, bio15 = precipitation seasonality (coefficient of variation),

bio16 = precipitation of wettest quarter, bio17 = precipitation of driest quarter, bio18 = precipitation of warmest quarter, bio19 = precipitation of coldest quarter.

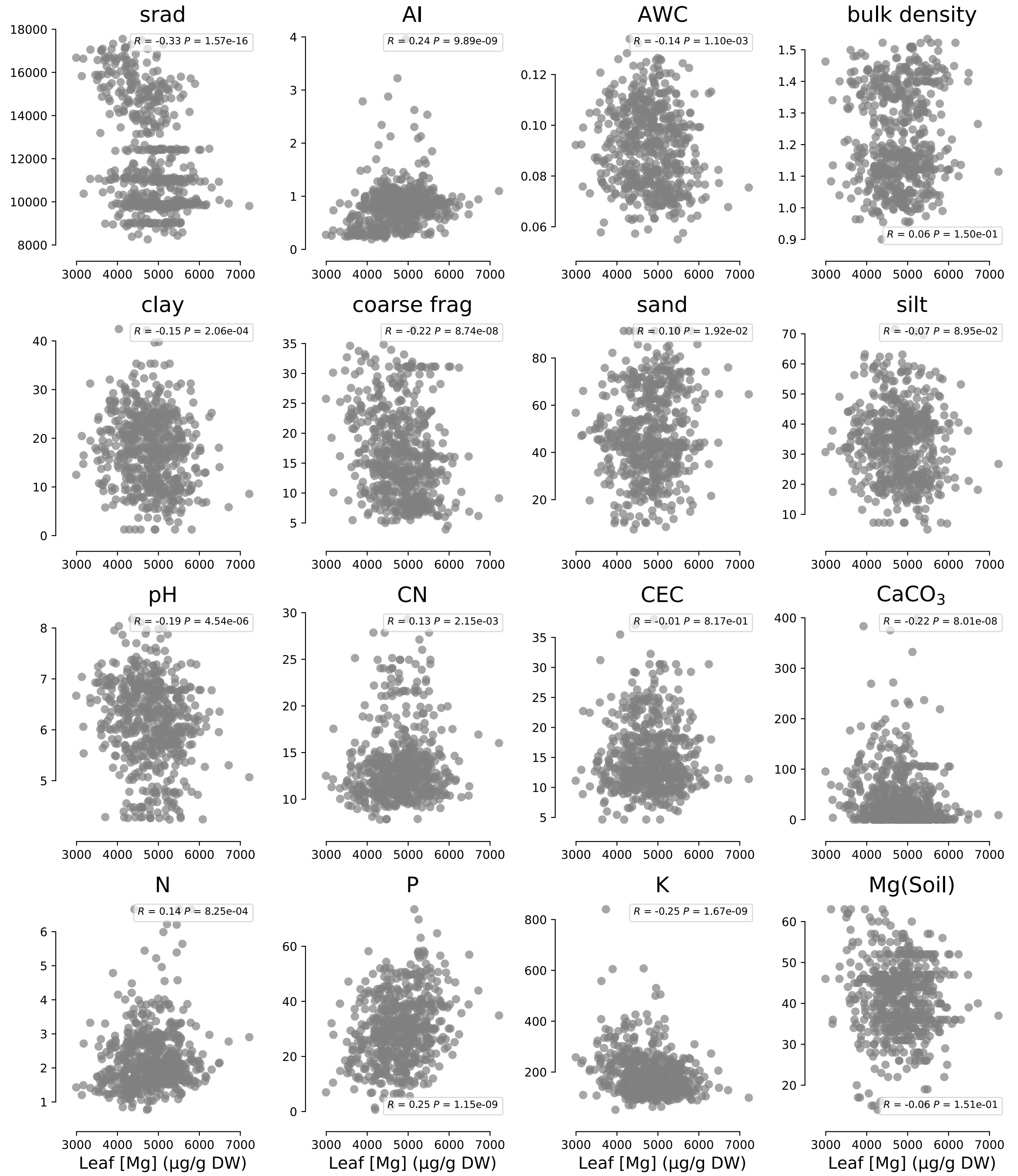

Fig. S3. Correlation between leaf Mg and other variables.

Leaf Mg concentrations are shown on the x-axis in µg/g of dry weight (DW). *R* = Spearman's rho, *P* = *P* value, n = 578 accessions. srad = solar radiation, AI = aridity index, AWC = available water capacity, CN = carbon nitrogen ratio, CEC = cation exchange capacity.

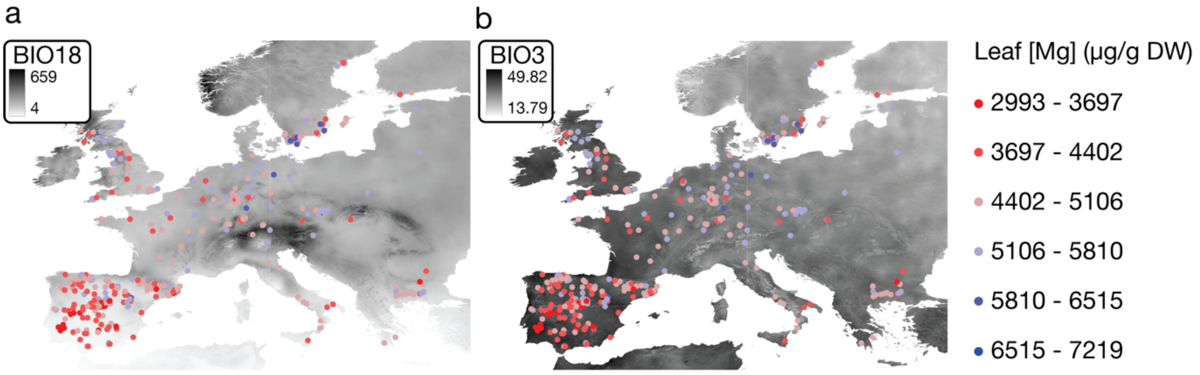

Fig. S4. Maps of the two most important predictive variables after solar radiation and BIO9.

Values are in mm for precipitation during the warmest quarter (BIO18) and in % for isothermality (BIO3). The color indicates leaf Mg content in µg/g of dry weight.

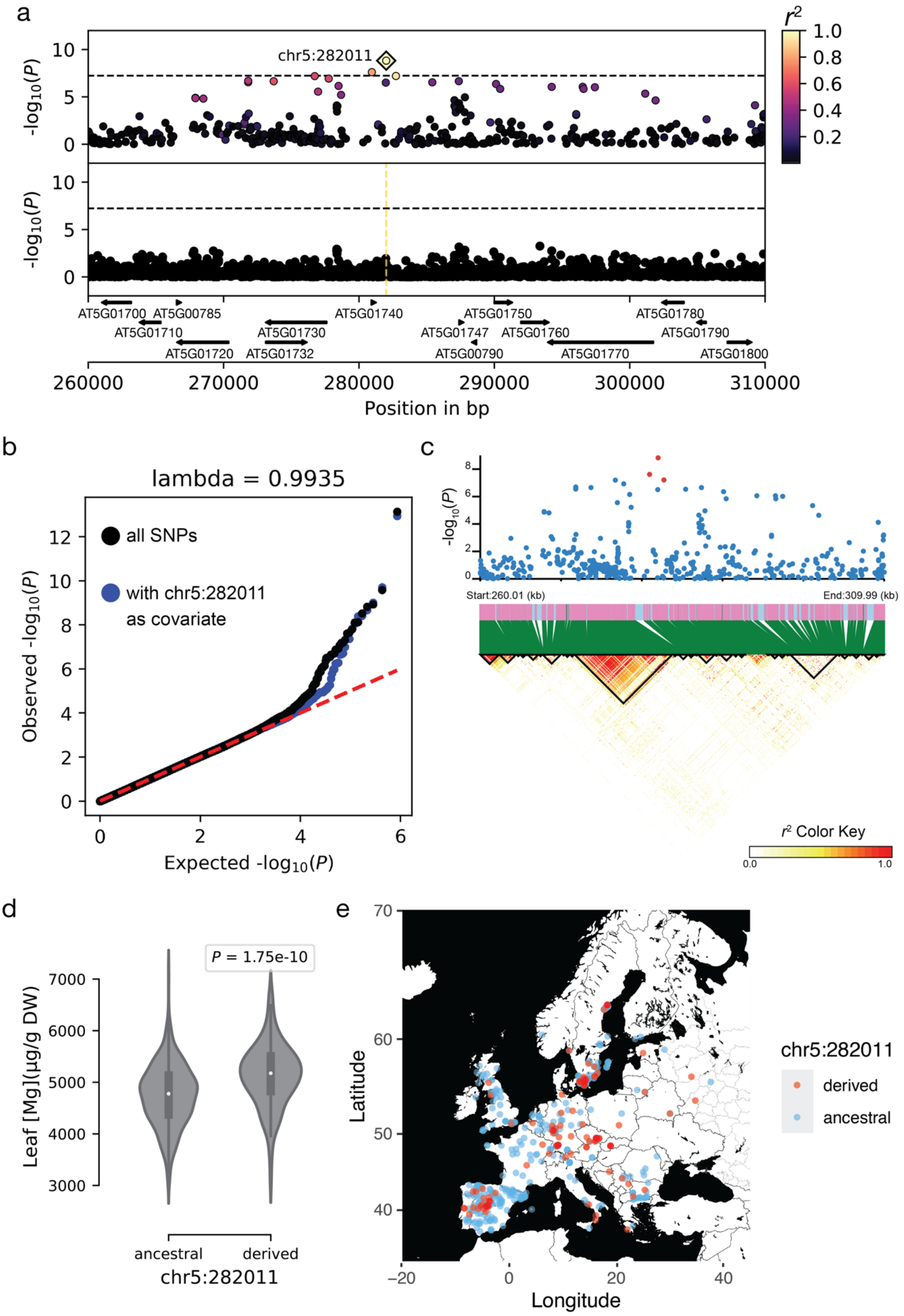
a
chr5:282011
-log10(P)
10
5
0
r2
1.0
0.8
0.6
0.4
0.2
AT5G01700 AT5G00785
AT5G01710
AT5G01720 AT5G01732
AT5G01730
AT5G01740
AT5G01747 AT5G01760
AT5G00790
AT5G01750
AT5G01770
AT5G01780
AT5G01790
AT5G01800
260000 270000 280000 290000 300000 310000
Position in bp
b
lambda = 0.9935
all SNPs
with chr5:282011 as covariate
Observed -log10(P)
Expected -log10(P)
c
Start:260.01 (kb)
End:309.99 (kb)
r2 Color Key
0.0
1.0
d
P = 1.75e-10
Leaf [Mg][μg/g DW)
7000
6000
5000
4000
3000
ancestral
derived
chr5:282011
e
Latitude
Longitude
chr5:282011
derived
ancestral

Fig. S5. One SNP (chr5:282011) explains the *RAPTOR1A* signal in the Eurasian panel.

a, Magnification of the peak at the *RAPTOR1A* region. The upper panel corresponds to the unconditioned GWAS. The lower panel corresponds to the GWAS after conditioning for the lead SNP (chr5:282011). Dashed horizontal lines correspond to the 5% Bonferroni-adjusted genome-wide significance threshold. Colors indicate linkage disequilibrium to the focal SNP (r^2) annotated with a diamond. *RAPTOR1A* is highlighted in red. **b**, Quantile-quantile plot showing the relationship between observed and expected associations with leaf Mg content. The red line indicates the expected relationship under the null hypothesis. Lambda corresponds to the genomic control. **c**, LD heatmap at the *RAPTOR1A* region. Red dots indicate genome-wide significant SNPs. **d**, Difference in leaf Mg content in µg/g of dry weight between the two chr5:282011 alleles. White dots of violin plots represent the median, boxes denote the 25th and 75th percentiles, whiskers extend from the box to the farthest data point lying within 1.5 times the inter-quartile range from the box. P = P value for MWW test. **e**, Geographical distribution of the chr5:282011 alleles in Europe.

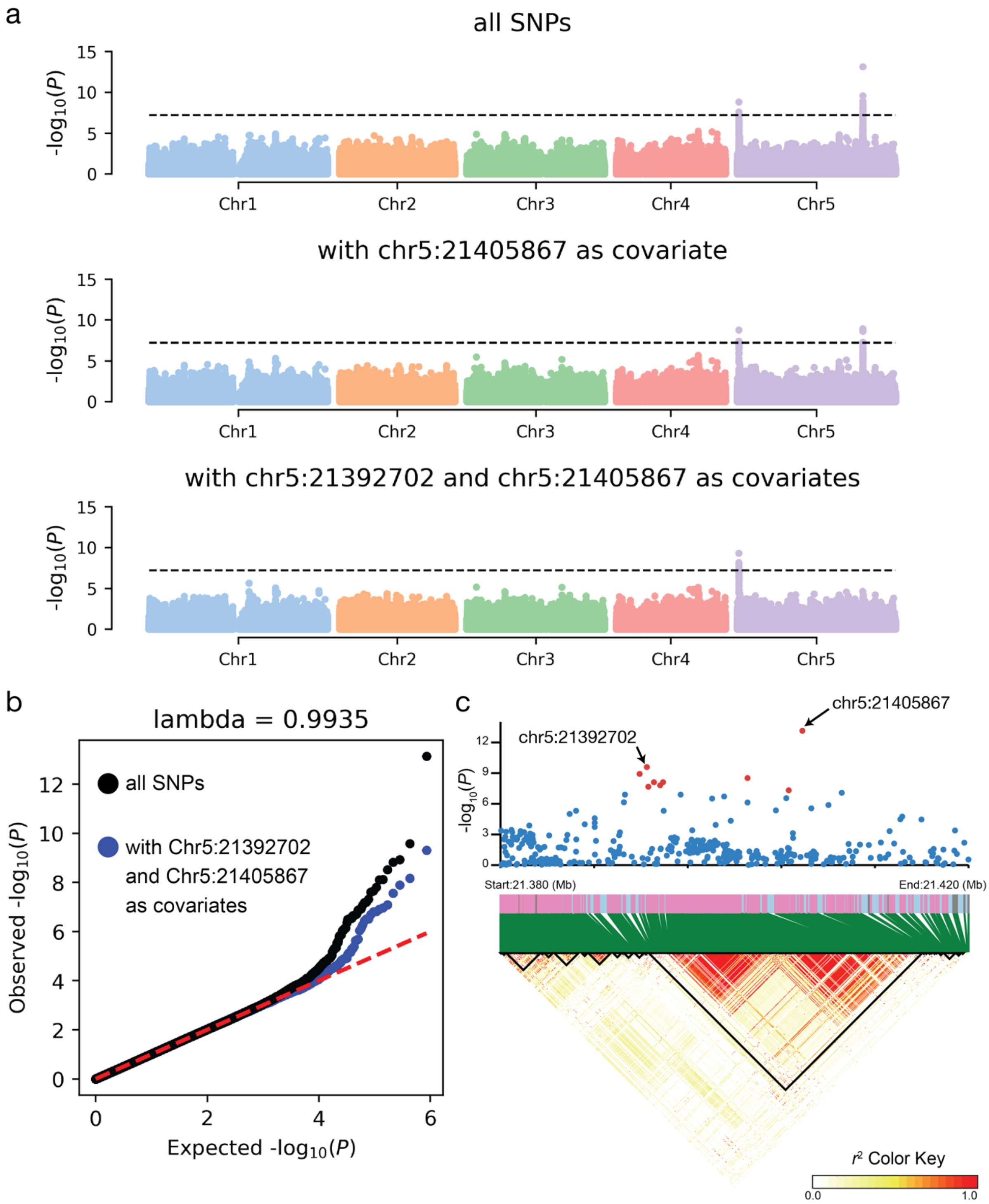

Fig. S6. Two independent SNPs explain the *MGR5* signal in the Eurasian panel.

a, GWAS for leaf Mg variation in the 1001 genomes panel. The upper panel corresponds to the unconditional GWAS. The middle panel corresponds to the GWAS after conditioning for the lead SNP at the *MGR5* region (chr5:21405867). The lower panel corresponds to the GWAS after conditioning for chr5:21392702 and chr5:21405867. **b**, Quantile-quantile plot showing the relationship between observed and expected associations with leaf Mg content. The red line indicates the expected relationship under the null hypothesis. Lambda corresponds to the genomic control. **c**, LD heatmap at the *MGR5* region. Red dots indicate genome-wide significant SNPs.

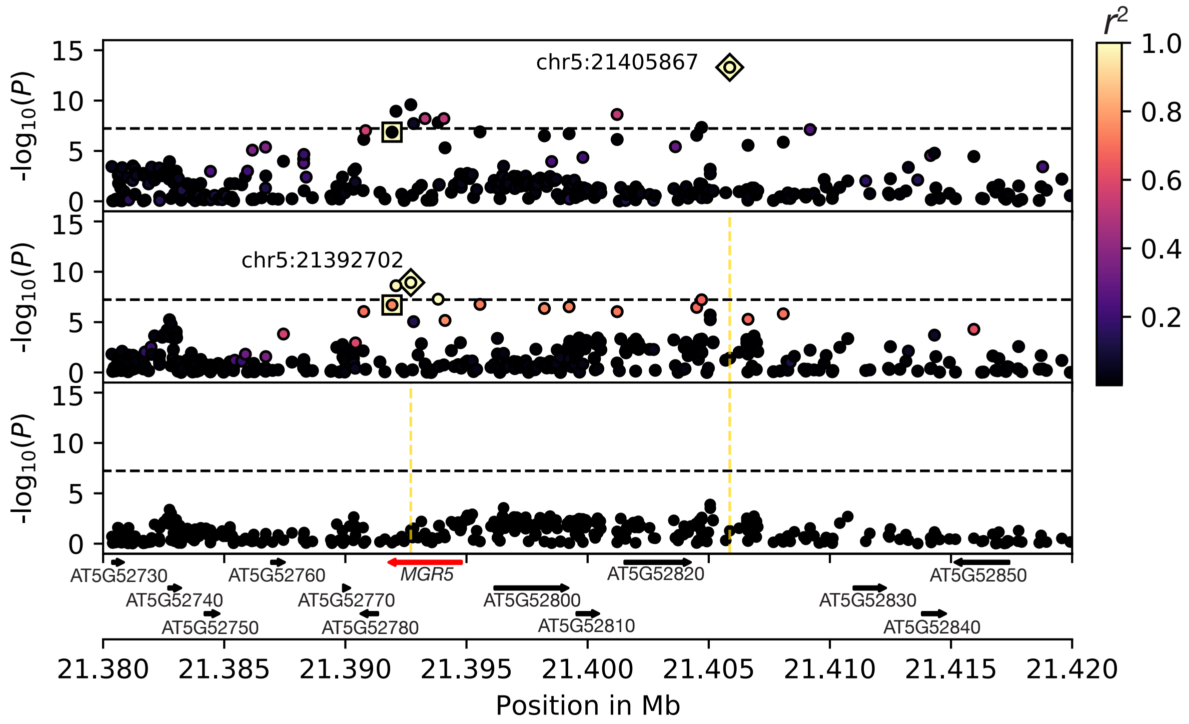

Fig. S7. Magnification of the peak at the *MGR5* region when including the *MGR5 S470fs* variant.

The upper panel corresponds to the unconditioned GWAS. The middle panel corresponds to the GWAS after conditioning for chr5:21405867. The lower panel corresponds to the GWAS after conditioning for chr5:21405867 and chr5:21392702. Dashed horizontal lines correspond to the 5% Bonferroni-adjusted genome-wide significance threshold. Colors indicate linkage disequilibrium to the focal SNP (r^2) annotated with a diamond. The *MGR5 S470fs* variant is annotated with a square. *MGR5* is highlighted in red.

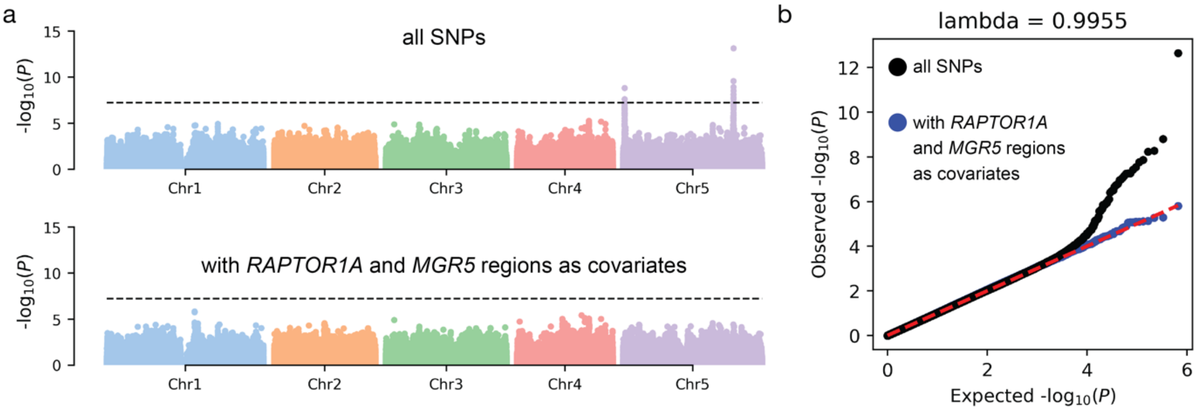

Fig. S8. The *RAPTOR1A* and *MGR5* regions explain most of the variation in leaf Mg content in the Eurasian panel.

a, GWAS for leaf Mg variation in the Eurasian panel. The upper panel corresponds to the unconditional GWAS. The lower panel corresponds to the GWAS after conditioning for the *RAPTOR1A* (chr5:282011) and the *MGR5* (chr5:21392702 and chr5:21405867) SNPs. **b**, Quantile-quantile plot showing the relationship between observed and expected associations with leaf Mg content. The red line indicates the expected relationship under the null hypothesis. Lambda corresponds to the genomic control.

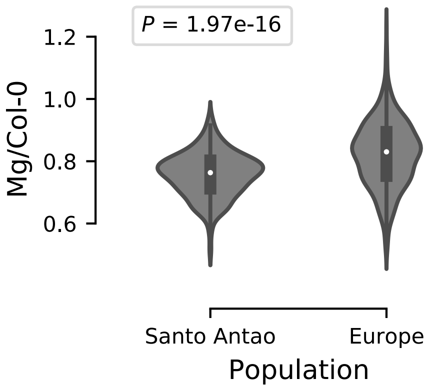

Fig. S9. Santo Antão accessions accumulate less Mg in leaf tissues compared to Eurasian accessions.

Violin plot showing the distribution of leaf Mg content in Santo Antão and Eurasian populations. Mg value for each accession is normalized to Mg values in Col-0. White dots of violin plots represent the median, boxes denote the 25th and 75th percentiles, whiskers extend from the box to the farthest data point lying within 1.5 times the inter-quartile range from the box. *P* = *P* values for MWW test.

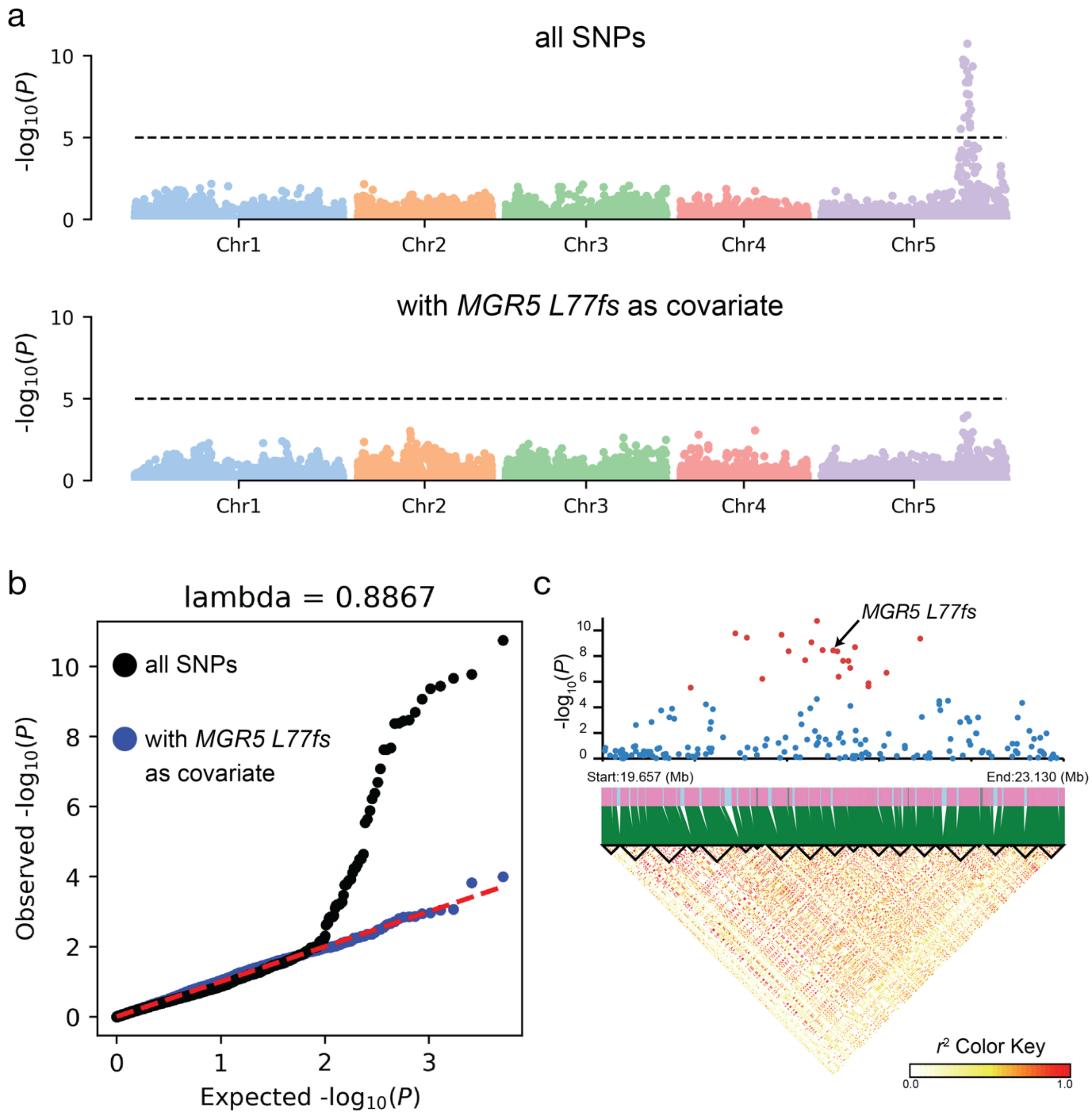

Fig. S10. Conditional GWAS for leaf Mg variation in Santo Antão.

a, GWAS for leaf Mg variation in the Santo Antão population. The upper panel corresponds to the unconditional GWAS. The lower panel corresponds to the GWAS after conditioning for *MGR5 77fs*. The chromosomes are color coded. **b**, Quantile-quantile plot showing the relationship between observed and expected associations with leaf Mg content. The red line indicates the expected relationship under the null hypothesis. Lambda corresponds to the genomic control. **c**, LD heatmap at the *MGR5* region. Red dots indicate genome-wide significant SNPs.

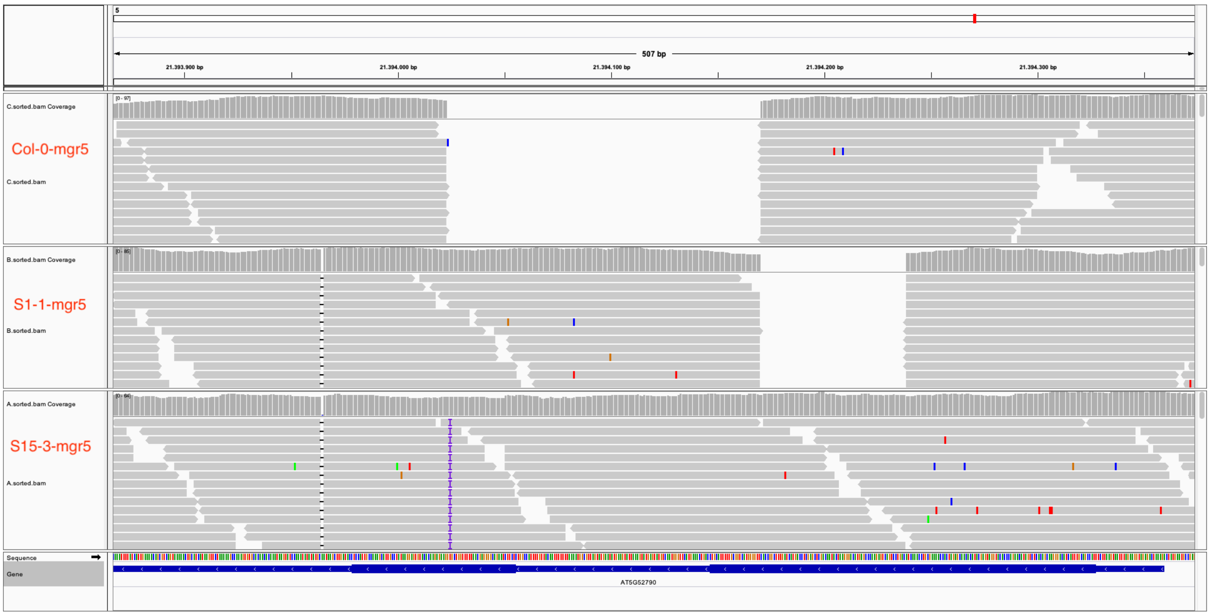

Fig. S11. ***MGR5*** **knock-out alleles generated in this study.**

IGV browser view of Illumina paired-end sequencing results showing the mutations at *MGR5* generated using CRISPR. Col-0 carries a 147 bp deletion, S1-1 carries a 68 bp deletion, and S15-3 carries a one bp insertion.
